# Quantification of Particle Filtration Using a Quartz Crystal Microbalance Embedded in a Microfluidic Channel

**DOI:** 10.1021/acs.langmuir.3c01331

**Published:** 2023-09-27

**Authors:** Siqi Ji, Ran Ran, Ilia Chiniforooshan Esfahani, Hongwei Sun, Kai-tak Wan

**Affiliations:** Mechanical and Industrial Engineering, Northeastern University, Boston, Massachusetts 02115-5005, United States

## Abstract

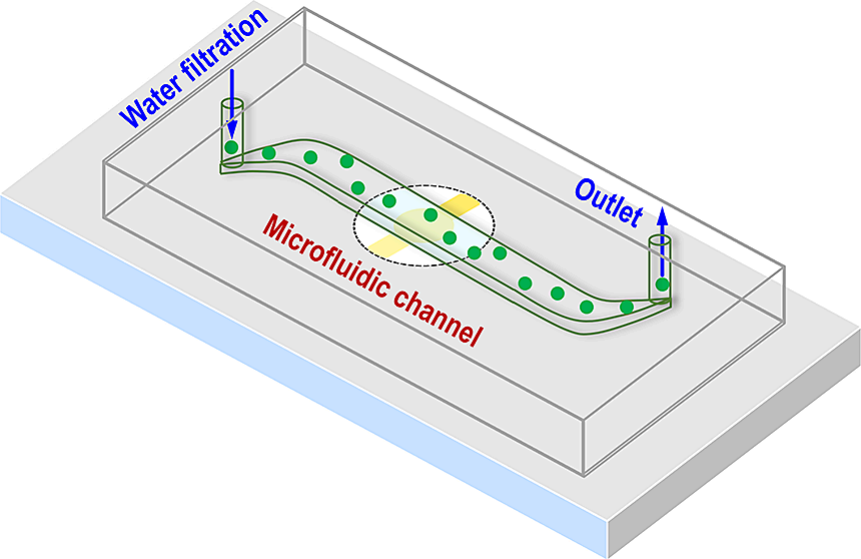

To quantify colloidal filtration, a quartz crystal microbalance (QCM) with a silicon dioxide surface is embedded on the inner surface of a microfluidic channel to monitor the real-time particle deposition. Potassium chloride solution with micrometer-size polystyrene particles simulating bacterial strains flows down the channel. In the presence of intrinsic Derjaguin–Landau–Verwey–Overbeek (DLVO) intersurface forces, particles are trapped by the quartz surfaces, and the increased mass shifts the QCM resonance frequency. The method provides an alternative way to measure filtration efficiency in an optically opaque channel and its dependence on the ionic concentration.

## Introduction

A filtration bed retains bacteria in liquid flow. The more particles trapped by the collector (e.g., sand grains), the higher the filtration efficiency. The underlying physics and mechanism of adhesion-detachment hold the key to a comprehensive understanding of water filtration. The classical colloidal filtration theory (CFT) discusses particle transport based on competition between convection and diffusion and has met many successes in the past decades.^1−4^

Most bacterial strains and model particles (e.g., polystyrene) as well as collectors carry negative surface charge. In the presence of an ionic solution, the surfaces attract cations or positive counterions and repel anions or negative co-ions in the solution, creating an electrostatic double layer (EDL). The net disjoining pressure at the particle-substrate interface is therefore a combined short-range van der Waals (vdW) attraction and long-range electrostatic repulsion. The resulting interaction and net force are conventionally and conveniently modeled by the Derjaguin–Landau–Verwey–Overbeek (DLVO) theory.^5−7^ Raising the ionic concentration effectively shields the electrostatic repulsion and thus enhances interfacial adhesion, raising the filtration bed efficiency. On the other hand, the increase in the flow rate over the collector surface boosts the hydrodynamic shear on particles adhered to the substrate. Beyond a critical flow rate, particles are detached and subsequently washed downstream. Additional parameters influencing filtration include stiffness, dimension, and geometry of the particles involved. A compliant particle is prone to deformation due to shear, and a larger particle experiences a larger drag force. The aspect ratio of a cylindrical particle as in common bacterial strains gives rise to differential shear and pressure distribution over the particle surface.^8−10^ To quantify filtration, adhered particles left on the collector surface are observed in situ by standard optical microscopy, provided the channel wall or collectors are optically transparent. It is possible to simultaneously monitor the number density and spatial distribution of the particles.

Microfluidic devices are ideal to quantify filtration efficiency due to their high sensitivity and small sample volume requirements. Here, we adopt a quartz crystal microbalance (QCM). As particles in a flow attach themselves to the sensor active surface, the net mass can be accurately measured.^11−16^ The device sets up a mechanical resonance of transverse shear wave with a frequency *f*_0_ on a planar surface of silicon dioxide.^17−20^ Once foreign materials or particles are trapped on the sensitive QCM area by physisorption or chemisorption, they move in sync with the substrate and thus experience a hydrodynamic drag due to the immersed aqueous medium. The additional inertia lowers the resonance frequency, and the negative frequency shift, Δ*f*, indicates the minute amount of adhered materials. The resolution can be as high as 10 ng/cm^2^.^21−25^ The QCM is remarkedly adapted to optically opaque channels or pipes.

It is noted that investigating live bacterial strain is technically challenging because they might die, multiply (i.e., growth), and aggregate during the experiments.^9^ It is therefore a common practice in the colloidal community to use polystyrene particles as model cells, because they have similar geometry, dimension, and mechanical properties, their surface chemistry can be modified to meet desirable experimental design, and no locomotion is possible.^6,9,26,27^

## Experimental Section

**Figure 1:**
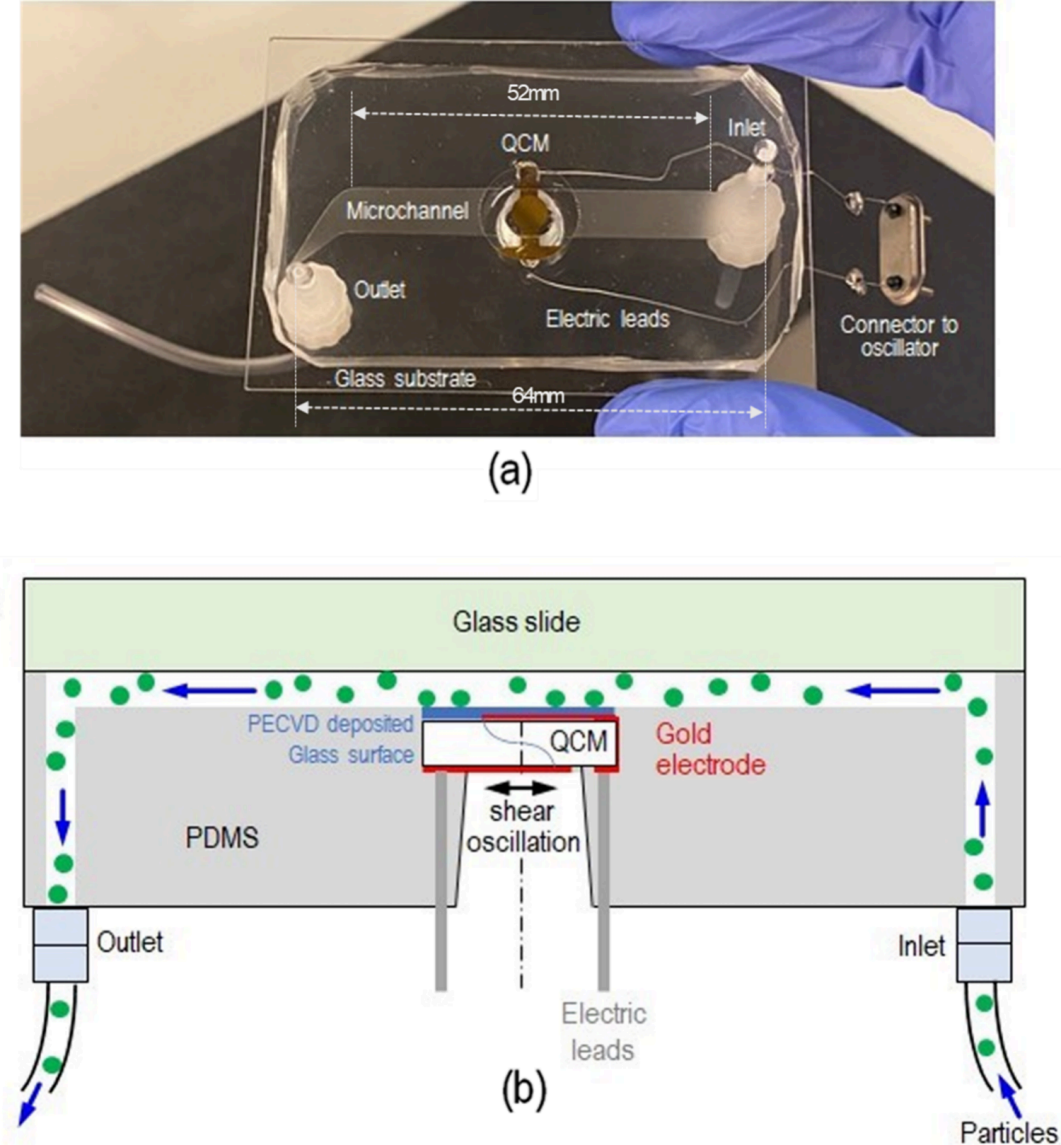
(a) QCM-embedded microfluidic device with the lead wires for frequency measurement and inlet and outlet for particle-laden flows; (b) schematic of the particle adhesion and detachment measuring mechanism by the QCM.

A commercial QCM with *f*_0_ = 10 MHz (Fortiming Corp., MA) based on an AT-cut quartz crystal is a 167 μm thick plate with both sides coated with a 10 nm chromium base layer and a top layer of 100 nm thick gold film to serve as the electrodes.^28,29^ An additional layer of 413 ± 32 nm thick SiO_2_ film measured by ellipsometry (alpha-SE ellipsometer, J.A. Woollam, NE) is then deposited on the 10 MHz QCM using plasma-enhanced chemical vapor deposition (PECVD) (PlasmaPro 100 PECVD, Oxford Instruments, MA) to form an active area. Surface characterization of the PECVD-deposited SiO_2_ film is shown in Figures S1 and S2 from the Supporting Information. The QCM-SiO_2_ plate is embedded in polydimethylsiloxane PDMS (Sylgard 184, Dow Corning) and bonded with a glass slide to form a microfluidic channel. Figure 1 shows the actual and schematic QCM-embedded microfluidic device with quick-turn tube connectors (McMaster-Carr, NJ) and the liquid flow direction.

**Figure 2:**
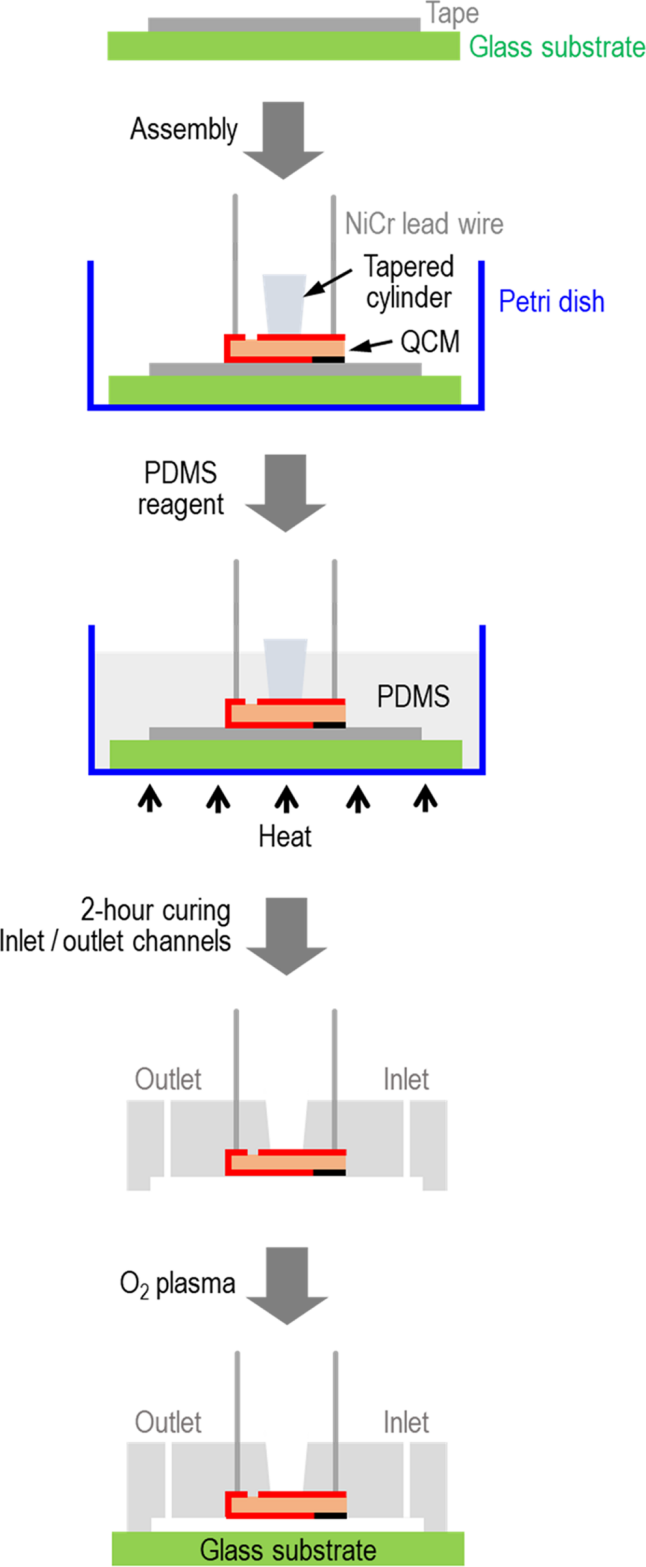
Step-by-step device fabrication.

Figure 2 shows the step-by-step device fabrication:

(i) A 100-μm-thick 3 M scotch tape serving as the mold is first mounted onto a glass slid. The tape is then tailor-cut into the designed microchannel with a rectangular cross-section.
(ii) Two nichrome lead wires are soldered onto the backside of the QCM sensing surface, and the assembly is pressed tightly on the tape-glass pair. The wires are designed to be connected to a frequency measurement system. A tapered 3D printed plastic cylinder is then mounted on the backside of the QCM to leave a window for subsequent QCM resonance. It is noted that the QCM cannot operate if it is entirely embedded in the PDMS block.
(iii) The QCM-tape-glass assembly is left on a glass Petri dish, and a mixture of PDMS base elastomer and curing agent with a mixing ratio of 10:1.2 is poured in and cured at 100 °C for 2 h.
(iv) The assembly is removed from the dish by peeling off the PDMS block from the glass slide and cutting off the extra surrounding edge. To construct the inlet and outlet at the opposite ends of the microchannel, two holes are punched on the PDMS block using a mechanical puncher (33-32-P/25, Integra, PA).
(v) The PDMS-QCM assembly and a glass slide are exposed to an O_2_ plasma etcher and treated for 1 min, before being pressed together to form a permanent bond. The interface is tested for leakage under pressurized steady flow.

**Figure 3:**
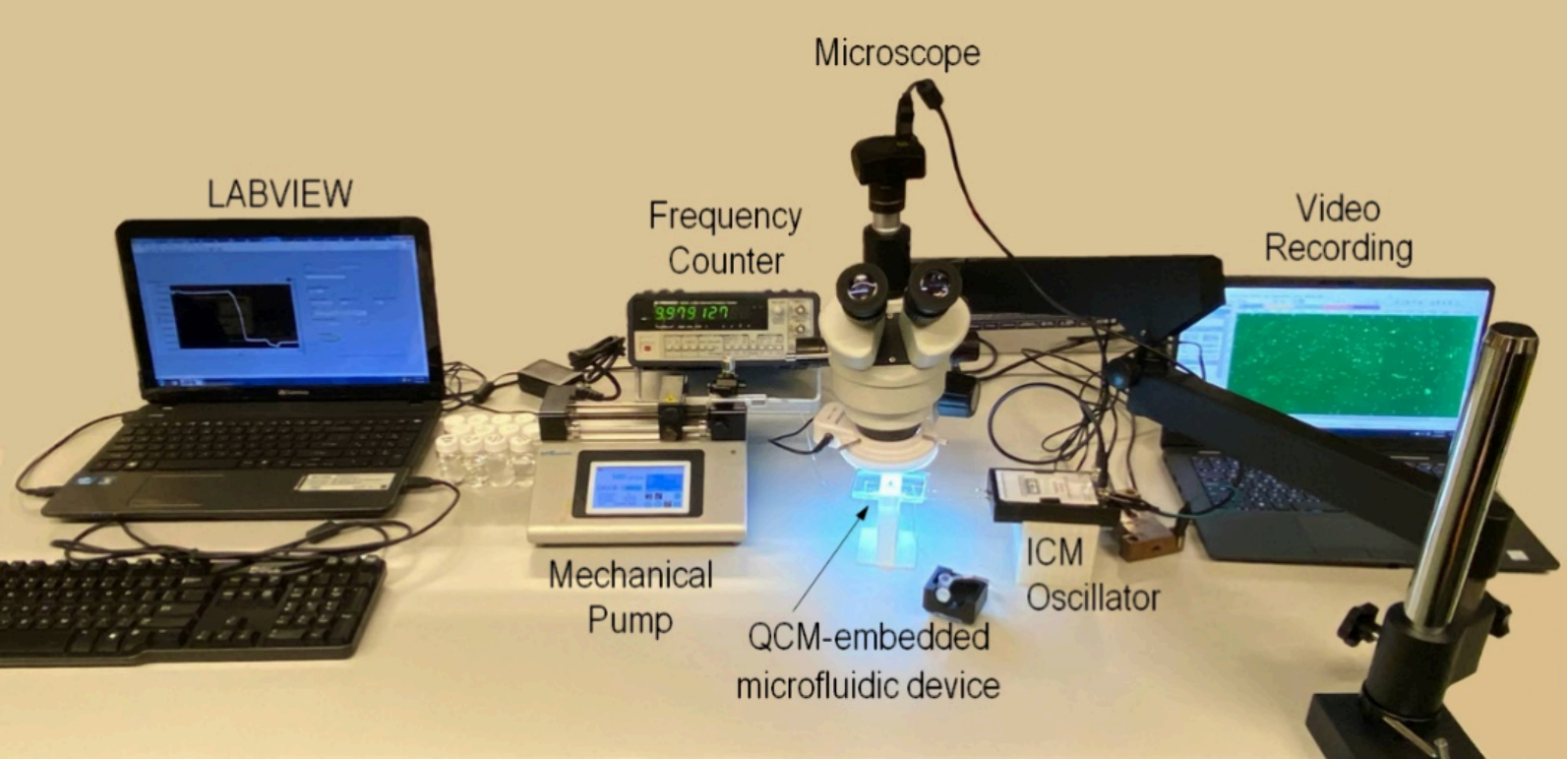
Experimental setup for particle adhesion-detachment measurement using the QCM sensor in a microfluidic device.

The microchannel has a rectangular cross-section of 100 μm × 6.5 mm and a length of 52.0 mm (i.e., an aspect ratio of 8.0). Uniform flow with *V* = 2.50 ± 0.10 mm/s is ensured (c.f. Figures S3 and S4 in the Supporting Information). The microfluidic device is mounted on a 3D printed plastics fixture when rubber tubing is attached to the microchannel inlet and outlet. An optical microscope (SM-8, AmScope, CA) held by a mechanical articulator is installed to monitor and video record the QCM surface. Figure 3 shows the assembly and associated accessories: (i) oscillator (35366-10, ICM, OK) to provide an AC voltage, (ii) frequency counter, and (iii) laptop with an in-house data acquisition system (Labview DAQ 2011). Polystyrene spheres (79633, Sigma-Aldrich., MO) with a diameter of *d*_p_ = ∼ 5 ± 0.1 μm and density of ρ_p_ = 1050 kg/m^–3^ serve as the particles. The particles are introduced into the relevant liquid with a particle number density of ρ ∼ 900 μL^–1^. The viscosity of the particle solution is measured and is found to be close to water in Figure S5 (Supporting Information). Each particle solution is freshly prepared and kept sonicating before injecting into the microchannel. The embedded QCM is functioning well as proven by the frequency spectrum in Figure S6 (Supporting Information), and no leakage is observed during all experiments.

The microchannel is first filled with potassium chloride solution KCl (aq) with a desirable concentration, *c*. To tare the QCM to achieve a robust baseline at fixed frequency, it is necessary to maintain the oscillation in stagnation solution for roughly 5 h. At *t* = 0 s, particle rich liquid flows into the channel at speed of *V* = 2.50 ± 0.10 mm/s or volume flow rate of *Q* = 100 μL/min for Δ*t* ∼ 300 s using a syringe pump (KdScientific, MA) installed at the outlet which draws the liquid into the channel. Particles are allowed to settle on the substrate in a stagnant liquid. Stochastically, some particles adhere firmly to the substrate, while the rest are either loosely attached or simply stay on the substrate with negligible interaction. Particle deposition on the QCM surface is monitored by the resonance frequency *f* as a function of time throughout the process, and the negative shift Δ*f* indicates the total mass of adhered particles. An optical microscope records the particle distribution. Once the QCM signal settles to a constant roughly over ∼10 min, particle free liquid is allowed to flow once again for ∼ 300 s to wash away any loosely bound particles. The QCM signal again is captured over the period and subsequent ∼1000 s. Measurement is repeated in KCl (aq) with an ionic concentration ranging from *c* = 3 to 30 mM, which is typical in underground water. The microchannel after each flow test is thoroughly cleansed using isopropanol followed by deionized (DI) water, followed by blowing nitrogen gas to dry. The device is then left in a dry environment overnight before the next measurement the following day. A control experiment is conducted using DI water to establish a baseline.

A MATLAB image processing routine (R2020a, MathWorks, MA) is implemented to count the particles trapped on the QCM surface post-mortem. Optical micrographs are converted into binary images by applying a critical threshold value of grayscale to outline the silhouette of particles.^9^ Based on the average number of pixels occupied by a single particle, multiparticle aggregates are differentiated and properly counted. The number of particles hereafter refers to the total number regardless of whether they are isolated or aggregated.

## Results and Discussion

### Frequency Response and Particle Counting

**Figure 4:**
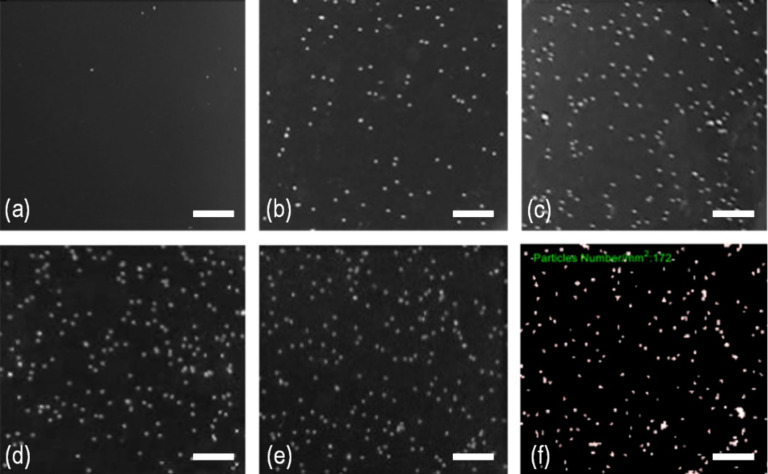
Particles adhered onto the QCM surface in KCl (aq) solution with a range of ionic concentration, *c*. Flow is from left to right. (a) DI water with number density ρ = 3 mm^–2^; (b) *c* = 3 mM and ρ = 66 mm^–2^; (c) *c* = 10 mM and ρ = 109 mm^–2^; (d) *c* = 20 mM and ρ = 138 mm^–2^; (e) *c* = 30 mM and ρ = 153 mm^–2^; (f) an example of particle counting for *c* = 30 mM by MATLAB routine showing the silhouette of isolated and aggregated particles. The scale bar is 50 μm.

Figure 4 shows the spatial distribution of adhered particles in a range of KCl concentrations. In the case of DI water (*c* = 0), virtually all particles are washed off with negligible entrapment. As the ionic concentration increases from *c* = 3 to 30 mM, more particles are trapped on the QCM surface raising the number density ρ as shown in Figures 4b–e. Figure 4f shows a typical black-and-white image converted from an optical micrograph by the MATLAB code. A few locations seem to be occupied by aggregates or multiple particles, which can be mostly differentiated by MATLAB with small error (<1%) in number counting.

**Figure 5:**
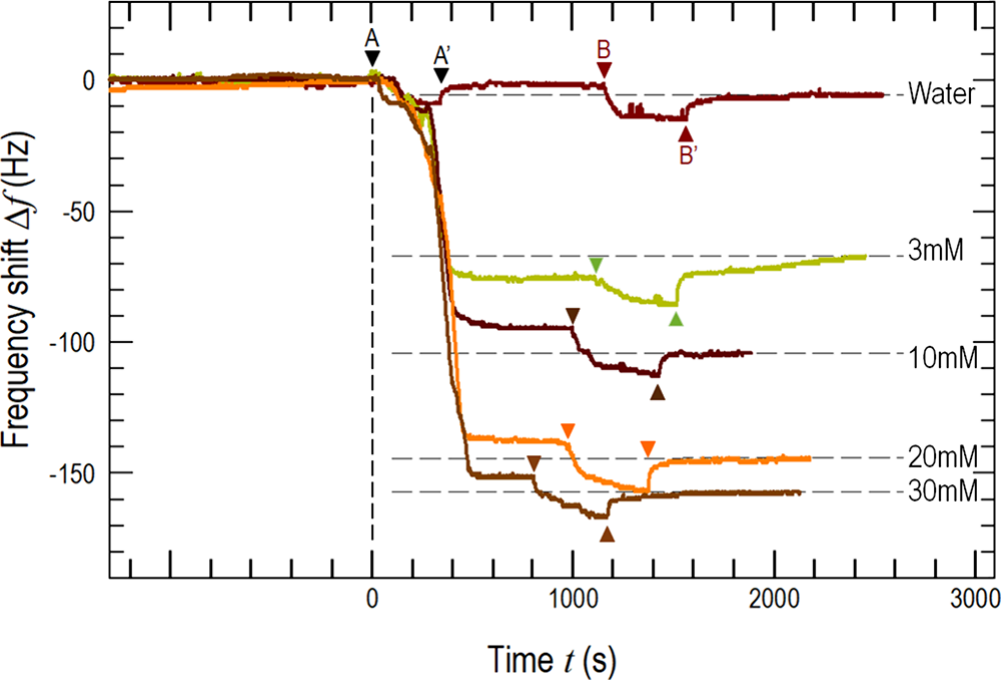
Real-time resonance frequency. Path A–A′ (DI water and electrolytes are marked with the same color of A–A′): At A, onset of particle-rich DI water/electrolytes flow into the microfluidic channel at 100 μL/min; at A′: pumping stops after 5 min; Path B–B′: At B, the onset of electrolyte with specific *c* flows down the channel at 100 μL/min to remove any trace of particles left in the system; at B′, pumping halts after 8 min. Final frequency changes with respect to different DI water/electrolytes are marked with dashed lines. In the case of *c* = 3 mM, the baseline rises gradually after B′ due to tiny bubbles generated during oscillation.

Figure 5 shows the frequency shift, Δ*f*, as a function of time after the onset of flow. In the case of DI water, Δ*f* (*t* < 0) = 0 represents the baseline noise. Particle-rich liquid starts to flow at A, reaches a steady flow, and halts at A′. Along AA′, the opposing hydrodynamic shear and particle adhesion cause Δ*f* to fluctuate and finally drop in an unsteady manner. After a finite relaxation time, most particles are washed off because of weak interfacial adhesion and Δ*f* (*t*) reaches a plateau with Δ*f* < 3 Hz, followed by a steady state. Flow resumes along BB′ but with the particle-free aqueous medium. Further loose particles are washed off the passage channel. A steady state once again is established, and Δ*f* =3Hzin essence. When the experiment is repeated in KCl (aq) with specific *c*, Δ*f* (*t*) shows a significant drop between steady flowsAA′ and BB′ compared to DI water. The more concentrated the electrolyte, the larger the drop in frequency Δ*f*, indicating more mass adhered to the QCM. It is noted that after the flow halts at B′, the leftover particles in the inlet tube are washed down and are likely trapped by the glass substrate, causing a small drop in frequency (6 ± 2 Hz).

**Figure 6:**
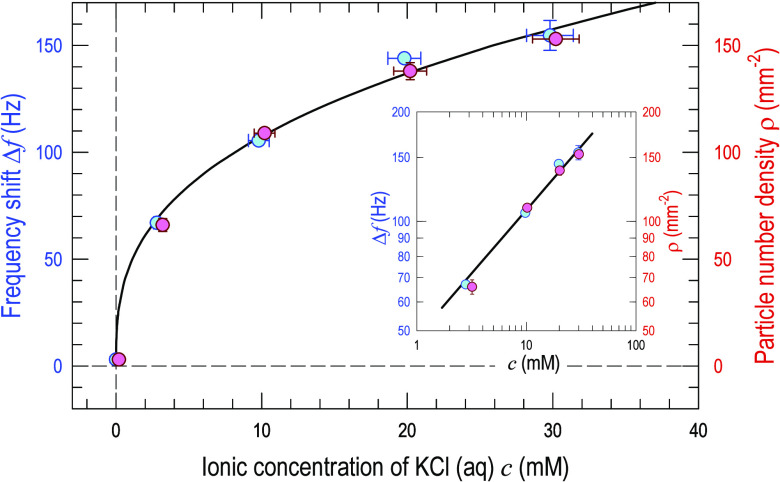
Frequency shift and number density of adhered particles as a function of the KCl (aq) concentration. The inset shows the same set of data in the log–log graph. A phenomenological equation, Δ*f*(*c*) is found by curve-fitting and is shown as curves. Note that the data points are slightly shifted to avoid overlap of the error bars.

**Figure 7:**
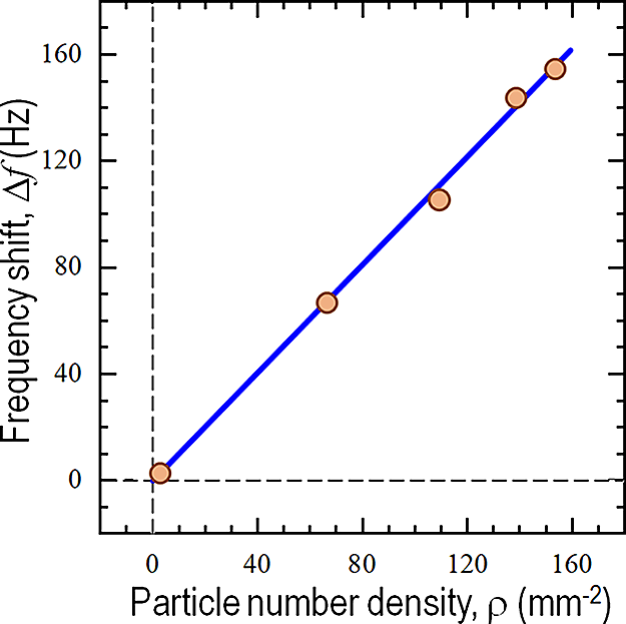
Comparison of QCM frequency shift as a function of measured particle density (circle) and predicted data by revised Sauerbrey theory (linear graph).

Figure 6 shows the frequency shift as a function of ionic concentration, Δ*f* (*c*). A phenomenological equation can be written for the absolute value of the frequency drop Δ*f* = (Δ*f*)_0_*c*^*n*^, with constants (Δ*f*)_0_ = 50.80 ± 5.46 and *n* = 0.33 ± 0.04, which is valid in the range of *c* = 0 to 30 mM. Similarly, the particle number density can be written as, ρ = ρ_0_*c*^*n*^ with ρ_0_ = 51.00 ± 4.34 and *n* = 0.33 ± 0.03. Figure 7 shows the frequency shift as a function of the number density ρ of particles stuck on the QCM, Δ*f* (ρ), which is a linear monotonic increasing function. One can therefore deduce from Figures 6 and 7 that the more concentrated the electrolyte, the more particles are trapped. QCM measurement is quantitatively consistent with the optical micrographs.

To quantify the adhered mass, the revised Sauerbrey theory^17^ assumes rigid particles spreading out uniformly over the QCM surface and adhering to the substrate by point contacts. The frequency shift is proportional to the *effective* mass of particles, Δ*m*_eff_ in grams, given by
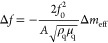
1with the fundamental resonant frequency *f*_0_ =10MHz, the sensing area of piezoelectric crystal *A* = 5.11 cm^2^, and the density ρ_q_ = 2.648 g·cm^–3^^17^ and shear modulus μ_q_ = 2.947 × 10^11^ g·cm^–1^·s^–2^^17^ of AT-cut quartz crystal. The effective total mass of particles stuck on the QCM, Δ*m*_eff_, is related to mass of single particle *m*_p_, density ρ_f_ ≈ 1000 kg.m^–3^ and dynamic viscosity η_f_ = 10 milli-poise of the aqueous medium, and the angular frequency, ω ≈ 2π × 10 MHz, of the QCM, and the total number of adhered particles *N* = ρ × *A*, given by,^30,31^
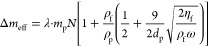
2For adhered particles subject to a rotational oscillation about the contact point, λ = 2/5.^32^ Using the numerical values of the physical parameters and substituting eq 2 into eq 1, a linear relation is found Δ*f* = 1.014 × ρ and is shown in Figure 7 with our measurements with reasonably good consistency. Note that the slow flow rate in this study leads to relatively weak hydrodynamic drag that is sufficient to detach the particles rather than causing any mechanical deformation on the rather rigid particles. In cases of very compliant particles or soft bonding or interfacial bonds with elastic spring nature,^32^ modifications to the classical Sauerbrey model is necessary but is beyond the present scope.

### Particle Number Density

**Figure 8:**
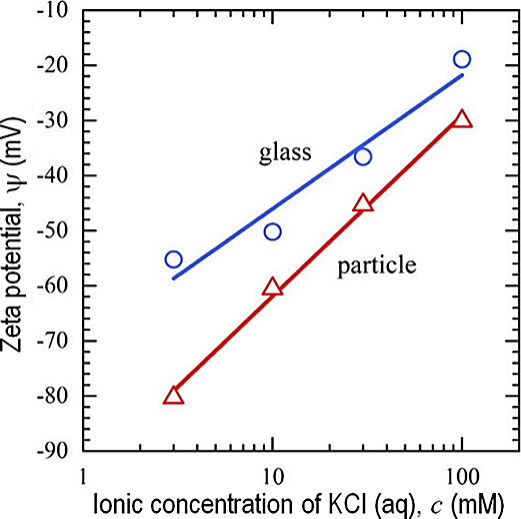
Zeta potentials of the glass substrate and polystyrene particle as functions of ionic concentrations, measured in ref (39). Numerical values used in our analysis are interpolations for 20 mM.

In the classical DLVO theory,^9^ energy density (J·m^–2^) between two planar surfaces separated by a distance *h* is given by
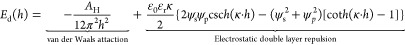
3The first term corresponds to vdW attraction. The Hamaker constant is given by  with *A*_w_ = 3.7 × 10^–20^ J for water,^33^*A*_s_ = 10.38 × 10^–20^ J for silica,^34^ and *A*_p_ = 6.88 × 10^–20^ J for the polystyrene particles,^35^ and with *A*_H_ = 0.9 × 10^–20^ J.^33,36^ The second term corresponds to EDL repulsion with permittivity in free space ε_0_ = 8.854 × 10^–12^ F/m,^37^ and dielectric constant of electrolyte or water ε_r_ = 80.1.^37^ The Debye screening length, κ^–1^, is a measure of the effective range of surface forces.^38^ A concentrated electrolyte leads to a short κ^–1^, and the particles stay closer to the substrate surface. Figure 8 shows the zeta potentials ψ_s_ and ψ_p_ of the glass substrate and polystyrene particle as functions of ionic strength.^6,39^ Numerical values of ψ_s_ and ψ_p_ for 3, 10, and 30 mM are measured in ref (39), interpolations are used for getting values for 20 mM.

**Figure 9:**
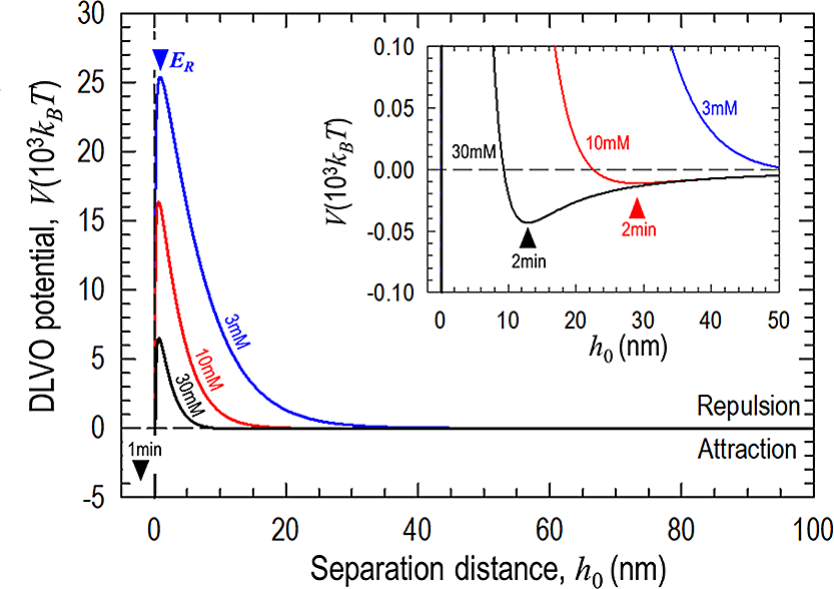
Surface energy density as a function of separation distance for KCl concentrations of 3, 10, and 30 mM. As electrolyte concentration increases, the repulsive energy barrier *E*_R_ diminishes, and the secondary minimum (2 min) is enhanced. The inset shows details of 2 min.

To account for a spherical particle interacting with an infinite planar substrate with a shortest separation *h*_0_, the total potential energy is found by integrating *E*_d_(*h*) (J·m^–2^) over the 2-D radial distance, *r*, from the axisymmetric axis (*r* = 0) to particle radius (*r* = *R*),^9^
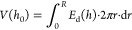
Since the intersurface force range is negligibly small compared to *R*, the upper integration limit can be set to +∞ to a high accuracy. The analytical expression is too long to be presented here but can be found in reference^40−43^. Figure 9 shows *V*(*h*_0_) for a range of *c*. In essence, the DLVO potential has a strong short-range attraction coined primary minimum (1 min) and a long-range secondary minimum (2 min), separated by a repulsion or energy barrier (*E*_R_).

In fresh water, the strong repulsion with a barrier of 4 × 10^4^*k*_B_*T* is too high for thermally excited particles at room temperature to overcome. The particles are repelled from the substrate, leading to Δ*m*_eff_ ≈ 0 and Δ*f* ≈ 0. In dilute solution (e.g., 3 mM), 2 min grows in strength and enhances adhesion, but the diminished repulsion barrier remains too high for the particles to penetrate. As *c* increases to ∼10 mM, 2 min is strengthened to ∼20 *k*_B_*T* trapping more particles. These particles are closer to the substrate surface due to the reduced Debye screening length. The corresponding *E*_R_ is now significantly reduced to stochastically allow particle passage to reach 1 min. Such particles now firmly adhere to the substrate and cannot be removed by flow at *V* ∼ 2.5 mm/s. As *c* increases to 30 mM, the apparent electrostatic repulsion further diminishes.

**Figure 10:**
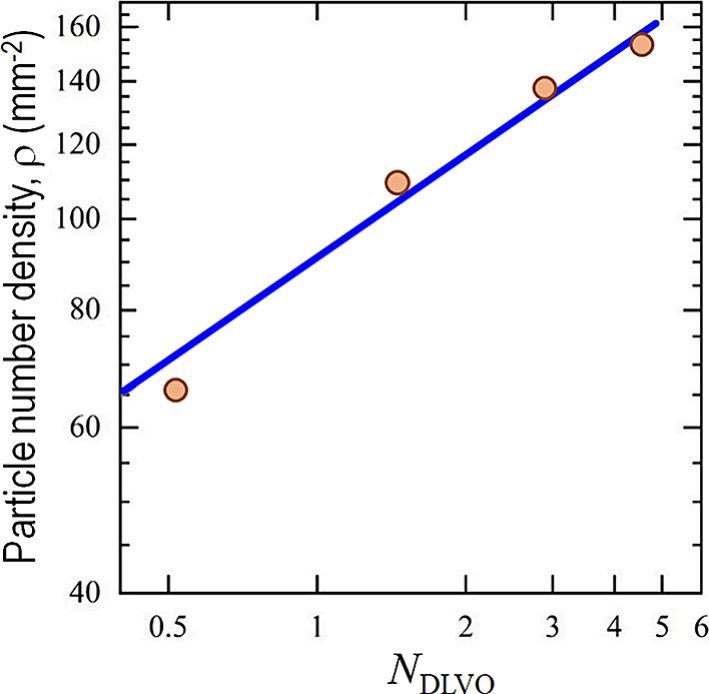
Number density of adhered particles as a function of *N*_DLVO_. Power-law fitting represented the correlation between the number density of adhered particles and *N*_DLVO_ (*R*^2^ = 0.98).

Colloid deposition on the surface primarily depends on the several factors, including the vdW attraction force characterized by *A*_H_, the EDL repulsion characterized by ε_0_ε_r_ψ_s_ψ_p_, and the range of electrostatic double layer characterized by reciprocal of Debye length.^43^ A dimensionless parameter gauges the ratio of vdW to EDL,^44^
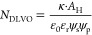
4that depends on ionic concentration. A large *N*_DLVO_ corresponds to a large adhesion, small repulsion, and more particles trapped on the substrate. Figure 10 shows ρ(*N*_DLVO_) based on measured ψ_s_ and ψ_p_. A phenomenological equation can be written as ρ = *C*_1_ × (*N*_DLVO_)^*C*_2_^, with the constants *C*_1_ = 90.96 ± 4.91 and *C*_2_ = 0.36 ± 0.04 determined by curve-fitting.

## Conclusions

A homemade microfluidic device with an embedded QCM is capable of the fast real-time characterization of colloidal particle deposition on glass in the presence of an electrolyte. Concentrated ionic solution enhances particle adhesion as a result of the reduced electrostatic double layer repulsion based on the classical DLVO model. Negative shift in the QCM resonance frequency yields the first estimation of the total mass deposited on the substrate by using the revised Sauerbrey theory and can be used as a measurement of the filtration efficiency. The dimensionless parameter *N*_DLVO_ is a practical gauge of particle adhesion or filtration efficiency. The device presents an alternative way of quantifying the filtration efficiency in an optically opaque pipe.

## Supporting Information Available

Dektak roughness profile of the PECVD-deposited SiO_2_ surface, average SiO_2_ film thickness measured by QCM, simulation results and microscopic photos indicating uniform flow in the microfluidic channel, measured viscosity of aqueous solution with polystyrene particles, and the embedded effect on the frequency response of the QCM-SiO_2_ device (PDF)
