# Supplementary material for: Quantification of Particle Filtration Using a Quartz Crystal Microbalance Embedded in a Microfluidic Channel
Source: Langmuir. 2023 Sep 27;39(40):14223–30. doi: 10.1021/acs.langmuir.3c01331 (PMC10620986; doi:10.1021/acs.langmuir.3c01331)
Supplement: Supplementary file 1 — la3c01331_si_001.pdf [file la3c01331_si_001.pdf]

Supporting Information

Quantification of Particle Filtration Using a Quartz Crystal Microbalance Embedded in a Microfluidic Channel

Siqi Ji, Ran Ran, Ilia Chiniforooshan Esfahani, Hongwei Sun, Kai-tak Wan*

Mechanical and Industrial Engineering, Northeastern University, Boston, Massachusetts, USA

*Corresponding author email: mktwan7@yahoo.com

Table of Contents

Figure S1 ____ 2

Figure S2 ____ 3

Figure S3 ____ 4

Figure S4 ____ 5

Figure S5 ____ 6

Figure S6 ____ 7

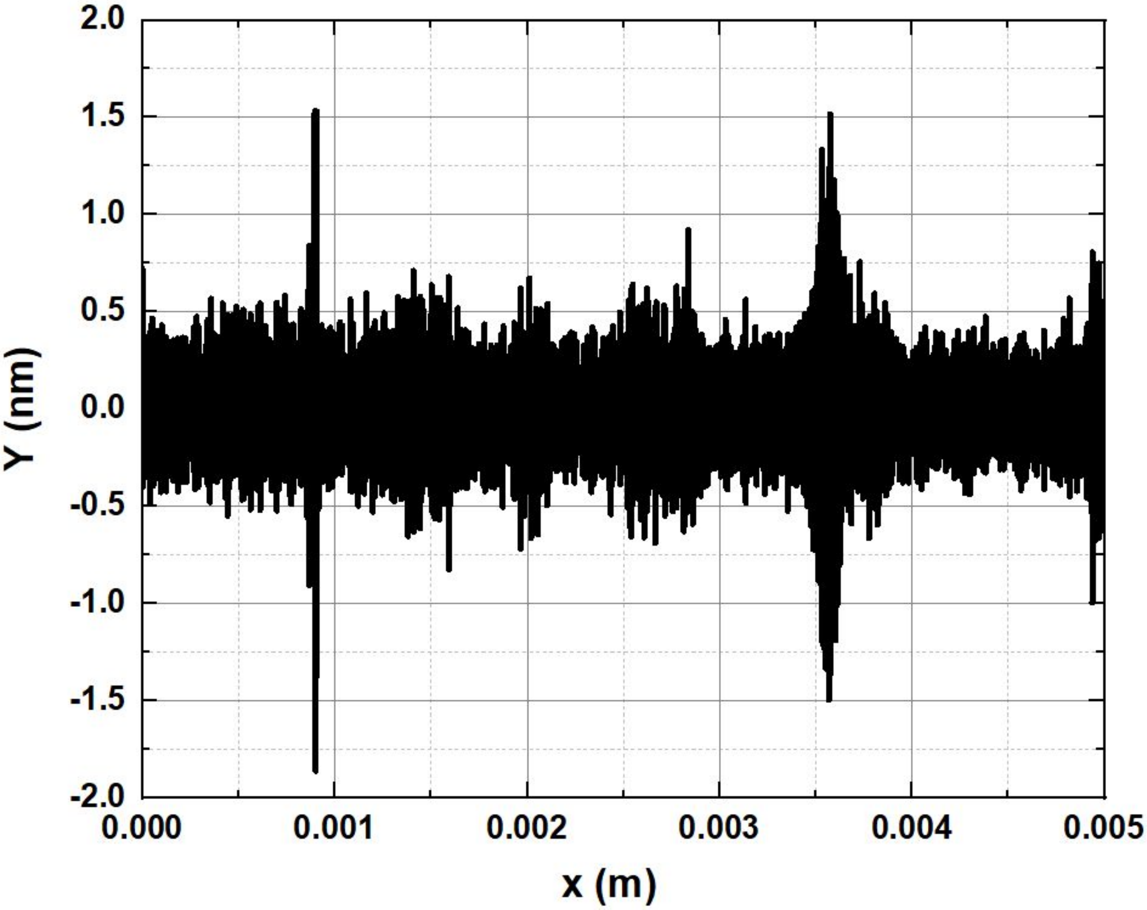

Figure S1. Typical surface scan on glass surface using Dektak surface profilometer (Bruker, MA) with a high pass FFT filter. Surface roughness is measured to be S_q = 0.17nm. Here x being length on sample surface and Y the local roughness.

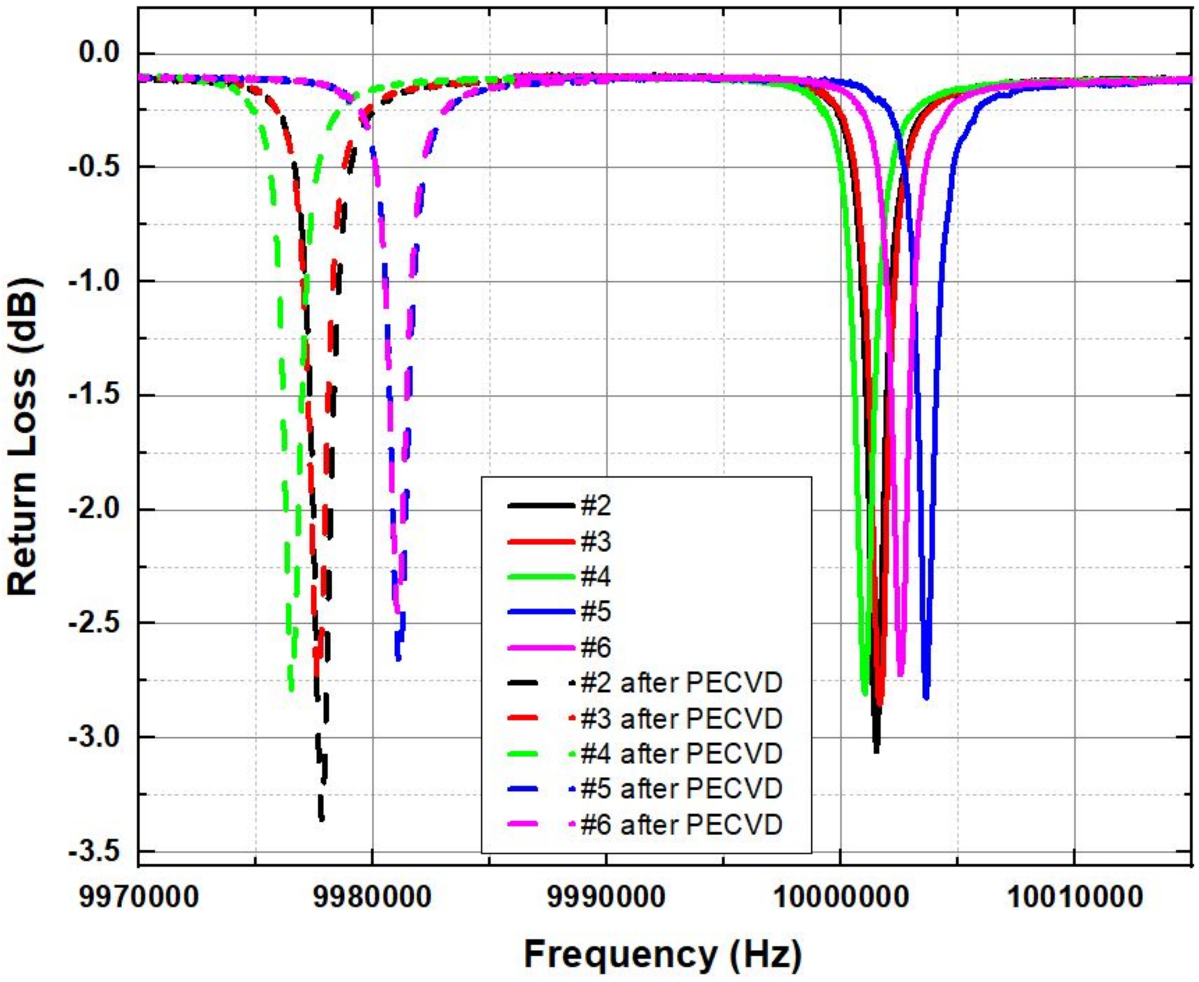

Figure S2. Resonance frequency shift after PECVD deposition (23310 ± 2126 Hz), indicating thickness of 389 ± 35 nm for this SiO_2 layer. Curves are labeled with #2 to #6 to indicate different QCM used in our measurement.

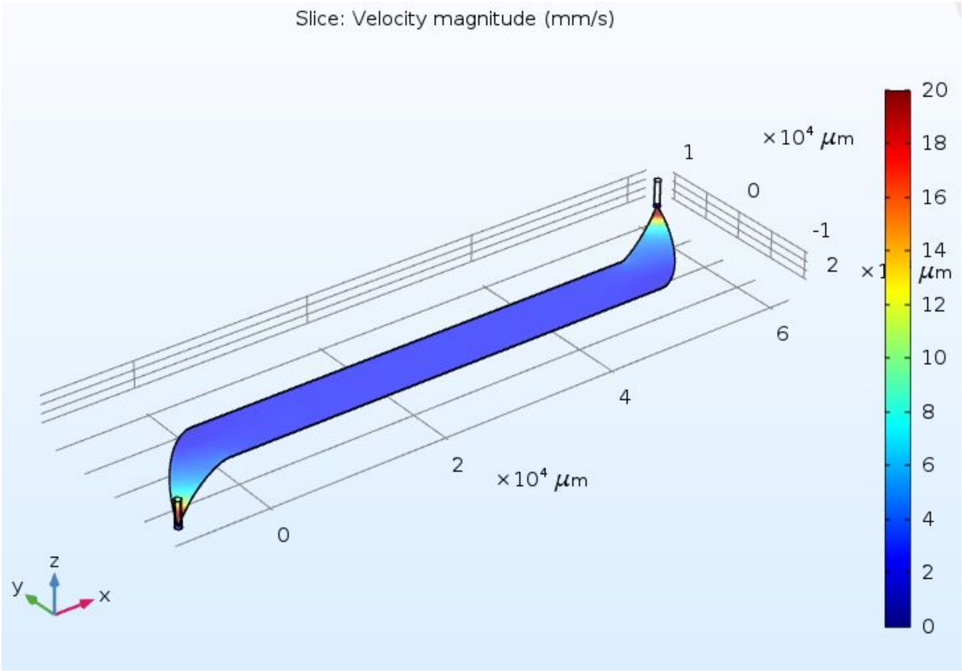

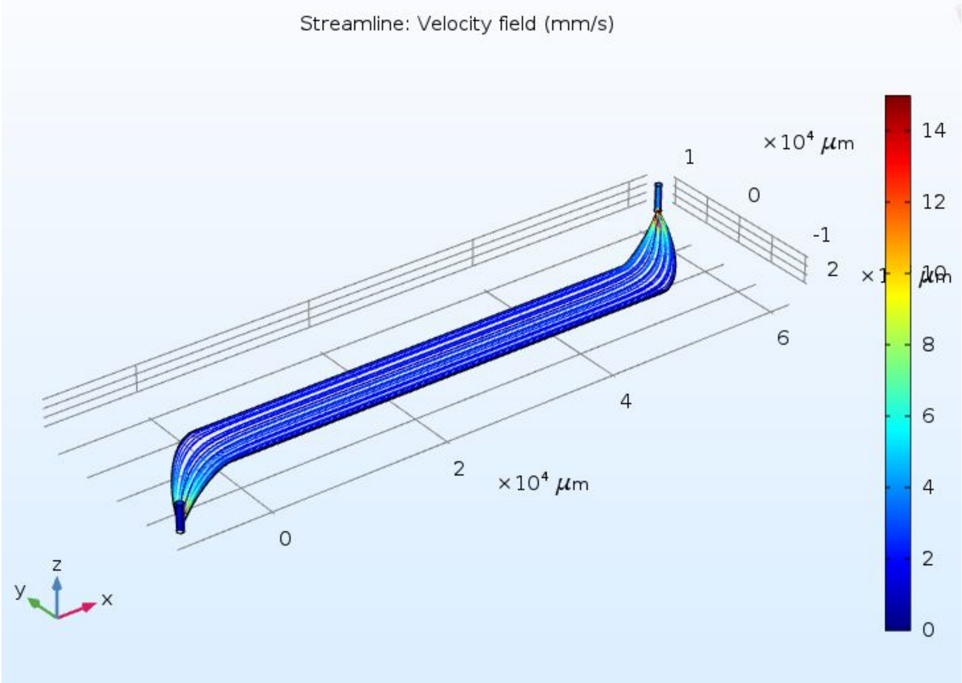

Figure S3. Flowrate at $V = 2.50 \pm 0.10$ mm/s (100 µL/min) running through the microfluidic channel with an aspect ratio of 8. Top: Velocity field at $z = 50$µm from the substrate; Bottom: Velocity streamline, generated by COMSOL.

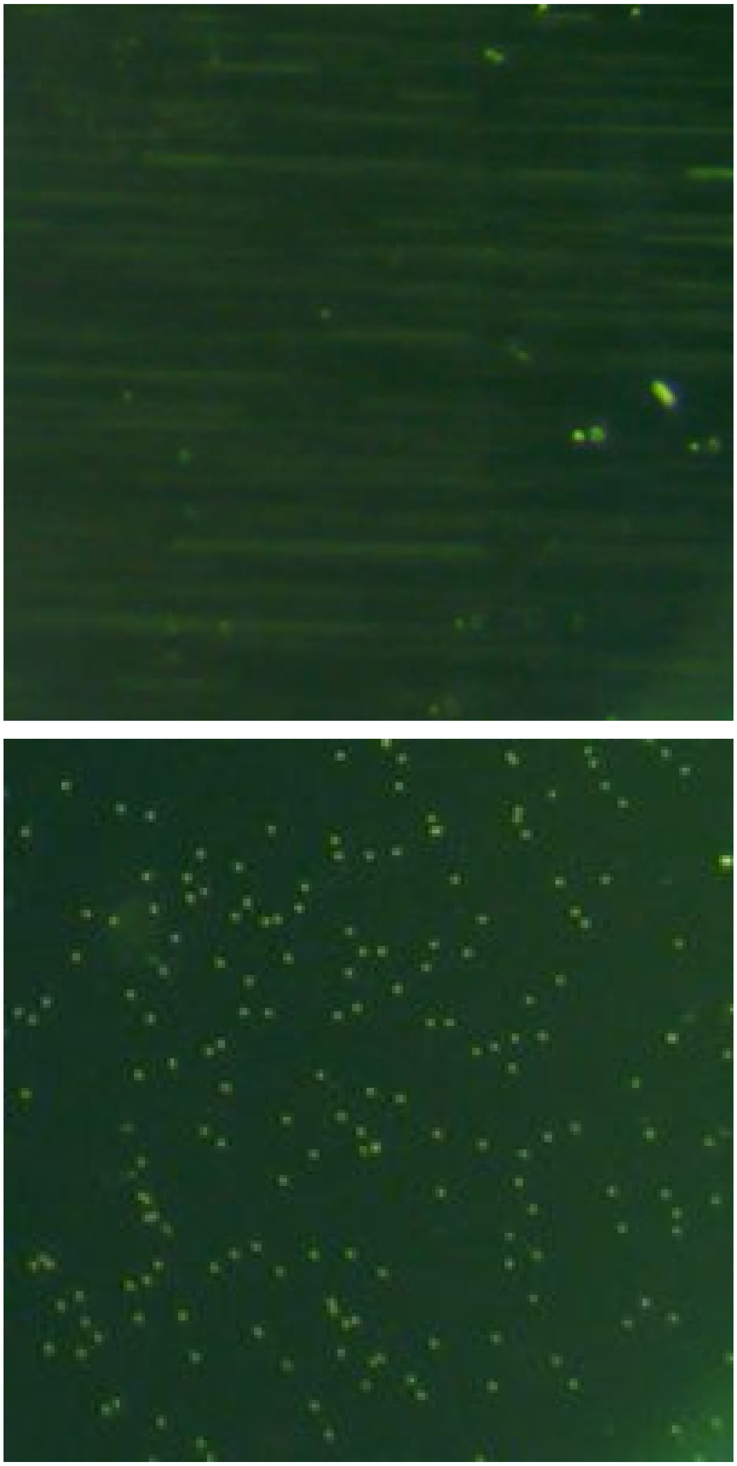

Figure S4. Top: **F**low in the microchannel with flow rate of $V = 2.50 \pm 0.10$ mm/s (100μL/min). Flow is shown to be quite uniform across the channel width. Bottom: Particle attached at quiescence.

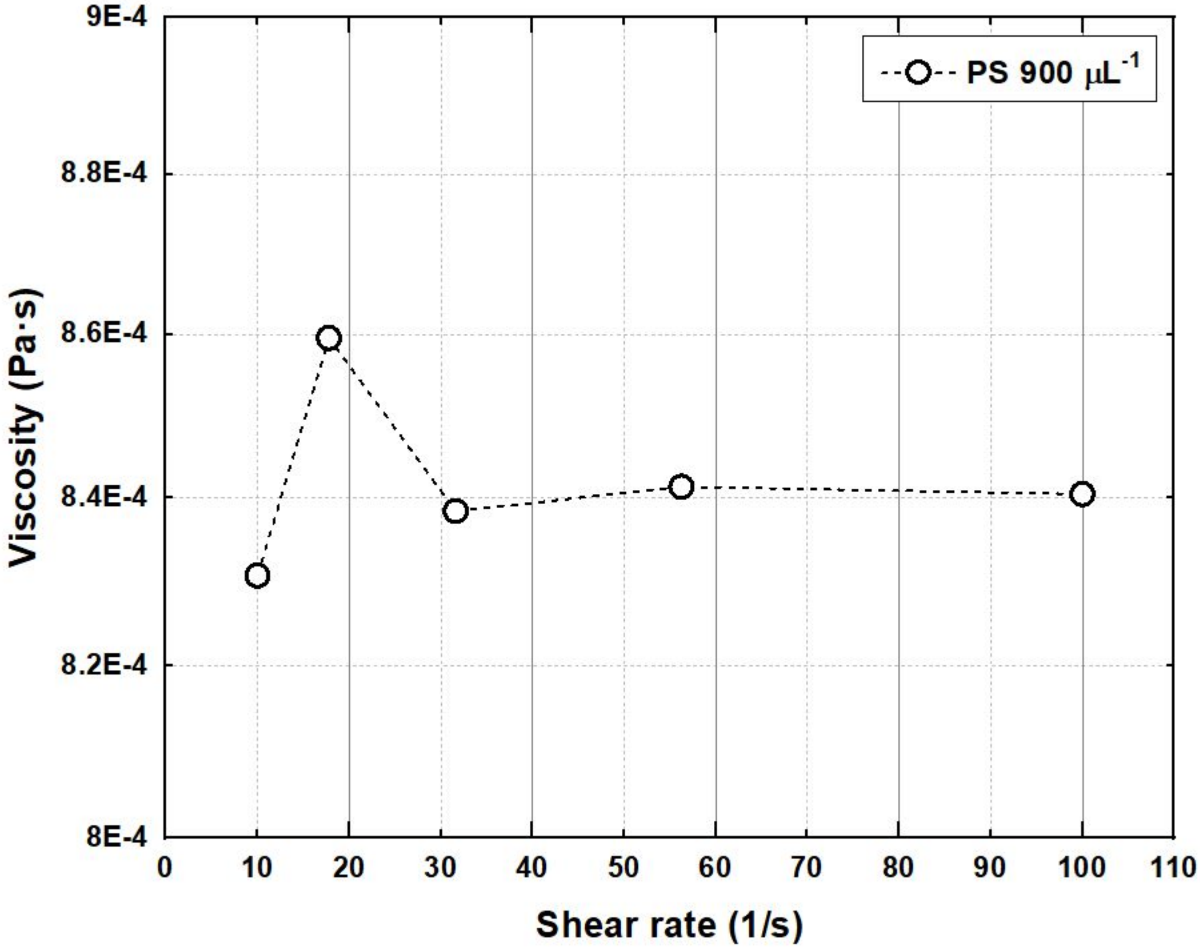

Figure S5. Viscosity of liquid containing polystyrene particles (900μL^{-1}) characterized by a rotational rheometer ARES-G2 (TA Instruments, DE) at 25°C under different shear rates (10 ~ 100 s^{-1}). The mean viscosity is measured to be (8.4 ± 0.095) × 10^{-4} Pa·s.

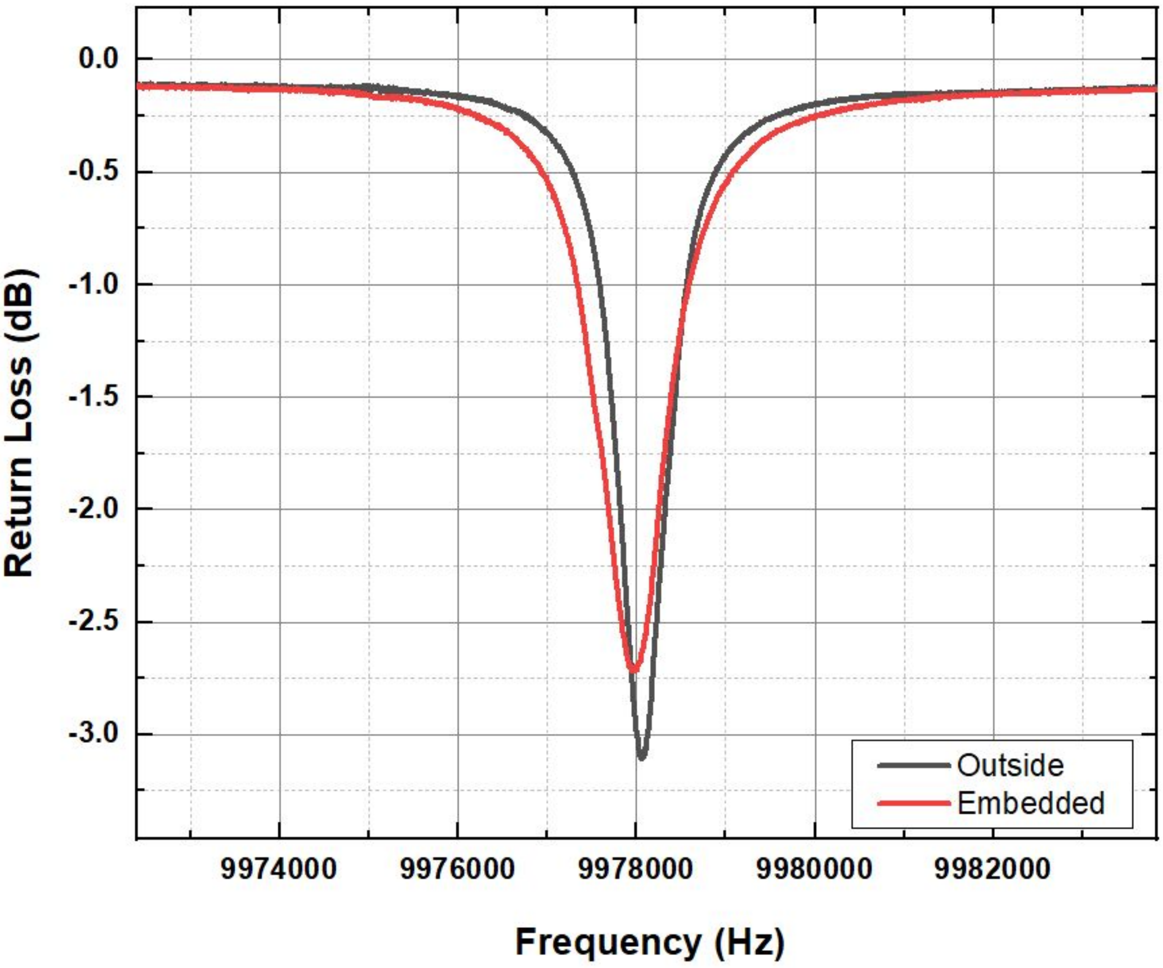

Figure S6. Frequency spectra for QCM-SiO_2 before and after embedded into PDMS microfluidic device. The peak and band width are essentially identical for our measurement.
